# Generation and characterization of a novel recombinant scFv antibody specific for *Campylobacter jejuni*

**DOI:** 10.1007/s00253-018-8949-x

**Published:** 2018-04-07

**Authors:** Ruramayi M. Nzuma, Fuquan Liu, Irene R. Grant

**Affiliations:** Institute for Global Food Security, School of Biological Sciences, Queen’s University Belfast, Belfast, Northern Ireland BT9 7BL UK

**Keywords:** *Campylobacter jejuni*, Recombinant scFv antibody, Immunomagnetic separation (IMS), Detection specificity, Phage display

## Abstract

*Campylobacter jejuni* is a leading cause of foodborne illness worldwide, mainly due to consumption and handling of contaminated raw chicken. Rapid detection methods for *C*. *jejuni* are vital for monitoring contamination levels in chicken products and reducing human Campylobacteriosis cases. The ‘gold standard’ culture-based method of *Campylobacter* detection takes 3–5 days and is too slow to permit effective intervention. Immuno-based methods are faster, but usually necessitate use of animals or hybridoma technology to produce antibodies; making them difficult and expensive to produce. Here, we report the generation and characterization of recombinant single-chain variable fragment (scFv) antibodies specific for *C*. *jejuni* cells, and evaluation of one scFv antibody for an immunomagnetic separation-quantitative PCR (IMS-qPCR) method to rapidly, sensitively, and specifically detect low numbers of *C*. *jejuni*. An scFv antibody phage-display library was constructed using spleen mRNA derived from a rabbit immunized with gamma-irradiated *C*. *jejuni* cells. This library was screened by surface biopanning against *C*. *jejuni* whole cells. Enriched clones were analyzed by enzyme-linked immunosorbent assay (ELISA). Two scFv antibodies that strongly and specifically recognized *C*. *jejuni* cell were expressed in *Escherichia coli*. Western blot analysis showed that one antibody, scFv80, was expressed as a soluble protein and retained its specific and strong binding to *C*. *jejuni* cells. This recombinant monoclonal scFv antibody was purified and used to covalently coat paramagnetic beads to be used for IMS-qPCR. The IMS-qPCR method was able to specifically and sensitively detect *C*. *jejuni* in mixed cultures within 3 h.

**Electronic supplementary material:**

The online version of this article (10.1007/s00253-018-8949-x) contains supplementary material, which is available to authorized users.

## Introduction

*Campylobacter*, especially *C*. *jejuni*, is one of the leading causes of bacterial foodborne illnesses worldwide (Silva et al. 2011; Tram et al. 2012). In the industrialized countries, *Campylobacter* spp. are the most common cause of foodborne illness in humans, ahead of *Salmonella* (Food Standards Agency 2009; Dominguez 2012). Data on Campylobacteriosis from developing countries is scarce, but there is a suggestion that the human burden due to *Campylobacter* infection is considerable (World Health Organization 2012; Platts-Mills and Kosek 2014).

Mishandling of contaminated raw chicken and consumption of undercooked poultry have been incriminated as the most significant source of *C*. *jejuni* infections for humans (Tam et al. 2009; Sheppard et al. 2009; Dominguez 2012). *Campylobacter* is a harmless commensal intestinal inhabitant of chicken (Doyle 1994), which makes it difficult to spot early infection of *Campylobacter* in a flock. It spreads quickly and 95% of a flock of 20,000 chickens can be infected by *Campylobacter* within 4 to 7 days after colonization of the first bird (Van Gerwe et al. 2009). Cross contamination of broiler carcasses can also occur in the slaughterhouse (Hermani et al. 2003; Tram et al. 2012). A significant correlation between the contamination of the broilers during rearing and the carcasses after processing has been observed (Hermani et al. 2003).

There is a need to monitor *Campylobacter* during the life of the chickens in broiler houses, to spot the infection as early as possible and to determine effectiveness of interventions. In the slaughter house, *Campylobacter* infection status needs to be checked in order to schedule flocks for slaughtering and prevent cross contamination (Tram et al. 2012). However, such monitoring has been hampered by the current ‘gold standard’ culture-based detection method, ISO/TS/EN 10272-2:2006 (International Standards Organization 2006). This method generally require 24–48 h enrichment culture before isolation of *C*. *jejuni* on selective culture media. Enrichment is followed by confirmatory methods such as biochemical and serological tests (Goossens and Butzler 1992), DNA/RNA hybridizations (Cudjoe et al. 1991; Ransom et al. 1994), enzyme-linked immunosorbent (ELISA) assay (Haas 1997), randomly amplified polymorphic DNA (RAPD) analysis (Mazurier et al. 1992; Hilton et al. 1997), polymerase chain reaction (PCR) or quantitative PCR (qPCR) assays (Giesendorf et al. 1992; Sails et al. 2003), and loop-mediated isothermal amplification (LAMP) of DNA (Yamazaki et al. 2009). Combination of culture enrichment procedure with these confirmatory methods leads to 3–5 day detection time, which is too long, bearing in mind the 4–7 day flock infection rate. By the time the results become available, the status of the flock may have changed, which may result in a false negative flock status. Another limitation of culture enrichment-dependent methods is their inability to detect viable but non-culturable (VBNC) *Campylobacter* cells that may be able to regenerate and become infectious again (Rollins and Colwell 1986; Silva et al. 2011). Direct detection of *Campylobacter* by PCR and qPCR methods without culture enrichment has been published (Rasmussen et al. 1996; Bang et al. 2001; Vondrakova et al. 2014). However, sensitive, specific, and rapid PCR detection can be hampered by inhibitors present in the crude sample matrix leading to false negative results (Tram et al. 2012). The other limitation of all the described culture-based methods is that only a very small portion (10–500 μl) of the considerable volume of biological samples (50–250 ml) is actually tested; this may also lead to false negative results, given that the abundance of *Campylobacter* spp. in biological samples may be low. Detection sensitivity of tests for *Campylobacter* spp. could be substantially improved if most of the *Campylobacter* cells in the large initial volume of sample could be concentrated into a small volume prior to testing.

To by-pass culture enrichment, immunomagnetic separation (IMS) methods have previously been developed using polyclonal (pAb) or monoclonal (mAb) antibodies to capture *Campylobacter* cells from milk, chicken meat, and chicken feces (Docherty et al. 1996; Lamoureux et al. 1997; Yu et al. 2001; Tram et al. 2012). IMS concentrates the target bacterial cells, thus reducing the sample volume, and removes sample constituents that may interfere with subsequent PCR; hence, the sensitivity of the assay is increased. However, production of appropriate antibodies is often time consuming, laborious, and expensive. pAbs can be only generated in limited amounts, dependent on animal size. The advent of hybridoma technology ushered in production of mAbs with defined antigen specificity and enables long-term antibody supply (Köhler and Milstein 1975; Lipman et al. 2005). However, the conventional hybridoma approach, which requires animal cell culture, is every expensive (Nelson et al. 2000). The clones produced also tend to lose antibody-secreting ability over time (Singh et al. 2010), limiting continuous supply.

Recombinant DNA technology has made it possible to generate monoclonal single-chain variable fragment (scFv) antibodies as recombinant proteins in *Escherichia coli* (Skerra and Pluckthun 1988). The scFv antibodies are a minimal form of functional antibodies which preserve the binding specificity of the parental IgG antibodies (Hagemeyer et al. 2009). A large repertoire of diverse scFv-libraries is constructed from RNA isolated from spleenocytes or the bone marrow of a host previously immunized with an antigen (Barbas et al. 2001). cDNA fragments coding variable heavy (V_H_) and variable light (V_L_) chains are amplified from the RNA by RT-PCR and randomly joined by overlap PCR. The resulting full-length scFv cDNA fragments are then cloned into a suitable expression vector to express the scFv antibodies. Their small size makes cloning into *E*. *coli* expression vectors easy, resulting in the ability to produce large quantities of monoclonal scFv antibodies quickly in bacterial cultures at a relatively low cost. The cloned fragments can be genetically engineered, if desired, e.g., to further optimize specificity and affinity (Hagemeyer et al. 2009).

Phage display is the most efficient system of expressing and screening recombinant scFv antibodies (Barbas et al. 2001). To this end, the full-length scFv cDNA fragments are cloned into phagemid vectors and introduced into *E*. *coli* cells to produce phage particles. The resultant display of the scFv antibody on the surface of the bacteriophage particle has been utilized as a means for effective screening for monoclonal scFv antibodies of desired specificity by phage biopanning (Barbas et al. 2001). Biopanning of scFv phage display libraries has been used to provide scFv antibodies against many antigens including foodborne contaminants *Listeria monocytogenes* (Paoli et al. 2007), staphylococcal enterotoxin B (SEB) (Singh et al. 2010), *Salmonella* Typhimurium (Meyer et al. 2012), and T3SS needle of *Vibrio parahaemolyticus* (Wang et al. 2014). However, to our knowledge, no recombinant anti-*Campylobacter* scFv antibodies have been reported to date.

In this study, recombinant rabbit scFv antibodies exhibiting specific binding to *C*. *jejuni* cells were constructed and selected by phage display technology. One of these anti-*C*. *jejuni* recombinant scFv antibodies was expressed in *E*. *coli* and purified. It was then coupled to tosylactivated paramagnetic beads to successfully capture *C*. *jejuni* cells in a specific and sensitive manner by IMS.

## Material and methods

### Preparation of irradiated *C*. *jejuni* cells

*Campylobacter jejuni* ATCC 29428 was cultured on Columbia Blood agar with 5% horse blood (Oxoid, Basingstoke, England) for 48 h at 42 °C under microaerophilic conditions. Cells were harvested and washed three times in phosphate buffer saline (PBS). The final pellet was resuspended in PBS to OD_600_ 1.0. Aliquoted cells were then gamma (γ)-irradiated with a dose of 10 kGy using a Gammabeam 650 cobalt irradiator (located at Agri-Food and Biosciences Institute for Northern Ireland, Newforge Lane, Belfast, Northern Ireland) and stored at − 80 °C to be used as the whole cell antigen (WCA) for antibody production and phage display biopanning purposes.

### Rabbit immunizations

Two New Zealand White rabbits (Q1 and Q2) were hyper-immunized with 10^9^ cfu/ml of γ-irradiated *C*. *jejuni* WCA. In total, 1 ml of WCA was mixed with 1 ml of mineral oil and administered to each rabbit in six injections over a 4-month period with 2-week intervals between the first and second injections, and then 4-week intervals subsequently. Blood was taken 10 days after each of the first five immunizations and titered by indirect enzyme-linked immunosorbent assay (ELISA) to determine presence of *C*. *jejuni*-specific antibodies*.*

### Total RNA extraction and cDNA synthesis

Fourteen days after the final immunization, rabbit Q1 was euthanized and exsanguinated. Spleen was harvested into RNALater (Thermo Scientific). Total RNA was extracted using 3 ml of TRI-reagent (Thermo Scientific) per 100 mg of tissue, following the manufacturer’s instructions. The RNA samples were then treated with DNase using TURBO DNA-free™ Kit (Ambion) to remove traces of DNA. Concentration of the RNA samples was quantified using NanoDrop™-2000, and 500 ng of RNA was checked by agarose gel electrophoresis to confirm no degradation of RNA. Five micrograms of total RNA purified from the rabbit spleen was used to synthesize first-strand cDNA using the SuperScript III First-Strand Synthesis System (Invitrogen) according to the manufacturer’s instructions.

### Generation of rabbit scFv cDNA library

First-strand cDNA derived from immune rabbit spleen was used to amplify scFv cDNA fragments with an overlap, 7-amino-acid (GGSSRSS), short linker (SL) sequence for diabody formation in two PCR steps using a procedure adapted from Barbas et al. (2001). In the primary PCR, cDNA fragments encoding the variable light (V_L_) chains (V_K_ and V_λ_) and the variable heavy (V_H_) chains were amplified separately. Using primers in Table 1, nine reactions were performed by pairing the three V_K_ 5′ forward primers with each of the three V_K_ 3′ reverse primers, to amplify the 350 bp V_K_ cDNA fragments; one reaction with V_λ_ 5′ forward primer RSCλ1 and V_λ_ 3′ reverse primer RJλo-B to amplify the 350 bp V_λ_ cDNA; and four reactions by paring each of the four V_H_ 5′ forward primers with the V_H_ 3′ reverse primer RSCG-B to amplify the 450 bp V_H_ cDNA. After PCR, V_L_ and V_H_ cDNA pools were separately made by combining the 10 V_L_ and 4 V_H_ PCR products. After primary purification using ethanol precipitation, the cDNA fragments encoding the V_L_ and the V_H_, respectively, were purified from a 1.5% agarose electrophoresis gel. These two fragments were mixed in equal molar ratios and used as the template in the secondary overlap extension PCR to amplify the full-length scFv cDNA fragments using primers RSC-F and RSC-R (Table 1). The assembled 800 bp full-length scFv cDNA fragment was purified by ethanol precipitation, resolved in a 1% agarose gel, and purified from gel bands.

**Table 1:** Details of PCR primers employed for construction of rabbit scFv fragments. Short linker sequences (GGSSRSS), underlined, are based on Protocol 9.7, Barbas et al. (2001)

Primer type	Primer name	Primer ID	Primer sequence
Variable Kappa light chain	Vκ5′ Forward	RSCVK1	5′ GGG CCC AGG CGG CCG AGC TCG TGM TGA CCC AGA CTC CA 3′
		RSCVK2	5′GGG CCC AGG CGG CCG AGC TCG ATM TGA CCC AGA CTC CA 3′
		RSCVK3	5′ GGG CCC AGG CGG CCG AGC TCG TGA TGA CCC AGA CTG AA 3′
	Vκ 3′ Reverse	RKB9J1o-B	5′ GGA AGA TCT AGA GGA ACC ACC TTT GAT TTC CAC ATT GGT GCC 3′
		RKB9Jo-B	5′ GGA AGA TCT AGA GGA ACC ACC TAG GAT CTC CAG CTC GGT CCC 3′
		RKB42Jo-B	5′ GGA AGA TCT AGA GGA ACC ACC TTT GAC SAC CAC CTC GGT CCC 3′
Variable Lambda light chain	V_λ_ 5′ Forward	RSCλ1	5′ GGG CCC AGG CGG CCG AGC TCG TGC TGA CTC AGT CGC CCT C 3′
	V_λ_ 3′ Reverse	RJλo-B	5′ GGA AGA TCT AGA GGA ACC ACC GCC TGT GAC GGT CAG CTG GGT CCC 3′
Variable heavy chain	V_H_ 5′ Forward	RSCVH1	5′ GGT GGT TCC TCT AGA TCT TCC CAG TCG GTG GAG GAG TCC RGG 3′
		RSCVH2	5′ GGT GGT TCC TCT AGA TCT TCC CAG TCG GTG AAG GAG TCC GAG 3′
		RSCVH3	5′ GGT GGT TCC TCT AGA TCT TCC CAG TCG YTG GAG GAG TCC GGG 3′
		RSCVH4	5′ GGT GGT TCC TCT AGA TCT TCC CAG SAG CAG CTG RTG GAG TCC GG 3′
	V_H_ 3′ Reverse	RSCG-B	5´ CCT GGC CGG CCT GGC CAC TAG TGA CTG AYG GAG CCT TAG GTT GCC C 3′
Overlap	Overlap Forward	RSC-F	5′ GAG GAG GAG GAG GAG GAG GCG GGG CCC AGG CGG CCG AGC TC 3´
	Overlap Reverse	RSC-B	5′ GAG GAG GAG GAG GAG GAG CCT GGC CGG CCT GGC CAC TAG TG 3′

pComb3XSS phagemid vector (obtained from Barbas lab, The Scripps Research Institute, CA; http://www.scripps.edu/barbas/) and the full-length scFv cDNA fragment were digested with *Sfi*I (Thermo Scientific), gel purified, and ligated using T4 DNA ligase, after dephosphorylation of the vector. Ligation product was purified by ethanol precipitation and resuspended in 15 μl water. Then 15 electroporations were performed; each transformed 1 μl of the purified ligation product into 25 μl *E*. *coli* ER2738 electrocompetent cells (Lucigen Corporation, USA). The transformed *E*. *coli* ER2738 cells were titered on Luria-Bertani (LB) agar plates with 100 mg/L carbenicillin to determine the number of independent clones in the library.

### Production of scFv displaying phage particles

To produce a library of phage particles displaying scFv, the transformed *E*. *coli* ER2738 scFv cDNA library was amplified and infected with helper phage M13K07 (Barbas et al. 2001). After overnight infection, the phage particles were harvested by polyethylene glycol (PEG) precipitation and resuspended in 2 ml 1% bovine serum albumin (BSA) in Tris-buffered saline (TBS) (50 mM Tris-HCl (pH 7.5), 150 mM NaCl), supplemented with 0.02% sodium azide (NaN_3_), to kill residual *E*. *coli* and prevent microbial growth, then filtered through a 0.2 μm syringe filter. The concentration of the phages in library was determined by titering, before storage at 4 °C. Prior to biopanning, the phage library was re-amplified as per Barbas et al. (2001). Only phage library freshly prepared on the same day was used for biopanning.

### Biopanning of phage displayed antibody library

The phage library was biopanned against irradiated *C*. *jejuni* WCA, in order to select anti-*C*. *jejuni* scFv displaying phages, that bound to *C*. *jejuni* cells with high affinity, using surface panning. Eight wells of a 96-well Nunc Maxisorp round-bottom microtiter plate (Thermo Scientific) were each coated with 100 μl *C*. *jejuni* WCA (1 × 10^9^ cfu/ml) in 0.1 M NaHCO_3_ buffer pH 8.6. The coated wells were blocked with 300 μl PBS containing 5% skimmed milk (5% PBSM) and washed twice with TBS with 0.05% Tween 20. Then, 100 μl freshly prepared starting phage library containing 6.8 × 10^7^ phages was added into each well and the plate was incubated at 37 °C for 2 h with gentle rocking. After decanting the phage solution, each well was washed five times with 200 μl TBS containing 0.5% Tween 20. The bound phages in each well were eluted using 100 μl of 100 mM Glycine–HCl pH 2.2 with 1 mg/ml BSA, and neutralized with 6 μl 2 M Tris (pH 9.1). The eluted phages were added into wells coated with *C*. *jejuni* WCA on the second plate and biopanning repeated as above (a procedure called double recognition, only applied to this first round biopanning). Eluted phage solutions were neutralized, titered to determine the output, and the rest of the phages amplified by infecting *E*. *coli* ER2738 cells before being rescued using helper phage M13K07 (Barbas et al. 2001). The amplified phages were titered and used as inputs for next round of biopanning as above, but with single recognition for all subsequent rounds. In total, six biopanning rounds were carried out against *C*. *jejuni* WCA to enrich the binding phages.

*C*. *jejuni*-binding by the enriched phages from rounds 3 to 6 was analyzed by ELISA. Wells of Nunc-Maxisorp microtiter plate were coated with irradiated *C*. *jejuni* WCA (1 × 10^7^ cfu/well) and 2.8 × 10^5^ phages were added into each well. Final anti-serum from rabbit Q1, parental phage that did not display a scFv, and unpanned phage display library were included as controls. Uncoated wells were included to assess the background binding. Bound phages were detected by horseradish peroxide (HRP)-conjugated anti-M13 monoclonal antibody (GE Healthcare) and its substrate 2,2′-azino-di[3-ethyl-benzthiazolin-sulfonate] (ABTS). The bound polyclonal antibody from the anti-serum was detected by HRP-conjugated anti-rabbit IgG (GE Healthcare) and its substrate TMB (3,3′,5,5′-tetramethybenzidine).

### Screening, selection, and characterization of scFv antibody-display phage clones

The phage clones eluted after round 6 biopanning were titered and colony PCR was performed to amplify the insert of scFv cDNA from 135 individual colonies using primers omseq (5′ AAGACAGCTATCGCGATTGCAG 3′) and gback (5′ GCCCCCTTATTAGCGTTTGCCATC 3′) which are located either side of the insert. PCR products were subsequently digested by *Mva* I and resolved in a 3% agarose gel. The clones were grouped according to their cleavage patterns.

The scFv antibodies were targeted against surface epitopes of *C*. *jejuni* whole cells, so representative clones from each group observed were analyzed for binding capacity to irradiated *C*. *jejuni* WCA by phage-binding ELISA. The individual phage clones were firstly amplified as described above. Concentration of amplified phage samples was quantified by quantitative PCR using primers pComb3xss_qPCR.F (TTTCCGTGTCGCCCTTATTC) and pComb3xss_qPCR.R (CCCAACTGATCTTCAGCATCTT) and Roche LightCycler ® 480 SYBR Green I Master. A 96-well Nunc-Maxisorp microtiter plate was coated with *C*. *jejuni* WCA (1 × 10^8^ cfu/well) and 2.8 × 10^5^ phages were used in each well. Bound phages were detected by HRP-conjugated anti-M13 monoclonal antibody (GE Healthcare) and its substrate ABTS. Phage clones which showed high binding activity to *C*. *jejuni* cells were also tested for reaction with non-irradiated *C*. *jejuni*, *Campylobacter coli*, and *Campylobacter lari* and three other unrelated foodborne pathogens, *L. monocytogenes*, *S*. Typhimurium, and *E*. *coli* by ELISA, as described above.

### Expression and purification of anti-*C*. *jejuni* scFv monoclonal antibodies

Clones scFv61 and scFv80, shown by ELISA to have the highest binding specificity, were chosen to express soluble anti-*C*. *jejuni* scFv antibodies. The phagemid DNA was purified from *E*. *coli* ER2738 and transformed into non-suppressor *E*. *coli* TOP10F′ cells (Invitrogen). Transformants were confirmed by colony PCR, as described above.

A single colony was inoculated into 10 ml pre-warmed super broth (SB) medium containing 50 μg/ml of carbenicillin and cultured overnight at 37 °C. The overnight culture was diluted 1:50 into fresh SB supplemented with 50 μg/ml carbenicillin and 40 mM MgCl_2_. The culture was incubated for 6 h at 37 °C with shaking at 250 rpm. Expression of the scFv antibodies was induced by adding isopropyl β-d-1-thiogalactopyranoside (IPTG) to a final concentration of 1 mM. The expression was induced for 18 h at 37 °C with shaking at 250 rpm. The culture was harvested by centrifuging at 3000 g for 30 min at 4 °C. The pellet was resuspended in 30–40 ml ice-cold extraction buffer containing 1× PBS, 1× Complete Cocktail Protease Inhibitor, 6.4 mM phenylmethylsulfonyl fluoride (PMSF), 5 mM MgCl_2_, 0.05 mg/ml DNase 1, and 0.05% β-mercaptoethanol. Cells were lysed by six French Press cycles at 1000 psi at 4 °C. The cleared lysate was centrifuged at 20,100 g for 30 min at 4 °C. The His-tagged scFv antibody in the supernatant was purified by adding 2 ml HisPur Ni-NTA Resin (Thermo Scientific), in the presence of imidazole. Binding was performed at 4 °C for 2 h with slow rotation. The beads were harvested by centrifuging at 2000 g for 5 min at 4 °C, washed twice in 50 ml ice-cold PBS containing imidazole for 10 min at 4 °C. The washed beads were resuspended into 2 ml of ice-cold PBS with high concentrations of imidazole and incubated for 15 min at 4 °C with gentle shaking. After a final centrifugation at 2000 g for 5 min at 4 °C, the supernatant containing the eluted antibody was aliquoted and stored at − 80 °C.

Presence of the scFv antibodies was checked by Western blotting. Protein samples were resolved by 15% SDS-PAGE before they were transferred onto Protran 0.2 NC nitrocellulose blotting membrane (Amersham). The membrane was blocked with a buffer consisting of 25 mM Tris pH 7.2, 0.15 M NaCl, 0.025% Tween-20, and 1% BSA for 1 h before being washed twice in TBS with 0.025% Tween 20. The His-tagged scFv was detected by incubating the membrane in 1:5000 dilution of India HisProbe-HRP (Thermo Scientific) in blocking buffer. After four washes with TBS containing 0.025% Tween 20, the membrane was incubated in SuperSignal West Pico Chemiluminescent Substrate (Thermo Scientific), exposed to X-ray film, and then developed.

### Characterization of soluble scFv80 antibody

The concentration of purified anti-*C*. *jejuni* scFv80 antibody was estimated by an ELISA assay, using His-tagged Cardiac Troponin I (cTnI) (GenScript) as the standard. Antigen-recognition capability and specificity of scFv80 for *C*. *jejuni* was then assessed by ELISA. A 96-well Nunc-Maxisorp microtiter plate was coated overnight at 4 °C with non-irradiated *C*. *jejuni* and five other non-target bacterial cells (10^8^ cfu/well). The plate was washed twice with 200 ml of TBS containing 0.05% Tween 20 (0.05% of PBS/Tween 20) and blocked with 200 ml of 1% BSA in PBS at 37 °C. After washing the plate twice with 0.05% of PBS/Tween 20, 100 μl scFv80 antibody (200 ng/ml) was added to each well, while PBS was added to control wells. The plate was incubated for 2 h at 37 °C with shaking and then washed three times with 0.05% of PBS/Tween 20. Bound scFv80 antibody was detected by adding 100 μl HisProbe-HRP conjugate (1:50,000 diluted in TBS containing 0.05% Tween 20) per well and incubating for 1 h at room temperature with shaking. Following four washes with 0.05% of PBS/Tween 20, 100 μl TMB(E) was added to each well, incubated at room temperature for 5 min, and then stopped with 25 μl 1 M H_2_SO_4_. Colorimetric signal was read at 450 nm using a plater reader.

### Development of an automated IMS procedure for capturing *C*. *jejuni* using anti-*C*. *jejuni* scFv80

BcMag™ long-arm and short-arm tosylactivated magnetic beads (10 mg each) (Bioclone Inc., San Diego) were covalently coated with 200 μg of purified scFv80 following the bead manufacturer’s instructions. To determine the capture capability and sensitivity of the coated beads, five ten-fold dilutions of non-irradiated *C*. *jejuni* cells (containing 10^7^–10^3^ cfu/ml) were processed by IMS, performed using a Dynal BeadRetriever instrument (Invitrogen). Each 1 ml dilution was mixed with 10 μl of coated beads (containing around 1.7 × 10^7^ beads) for 30 min, followed by two 1-min washes in PBS-0.05% Tween 20. The washed beads were resuspended in 100 μl TE buffer, pH 8.0, and heated at 99 °C for 10 min in a heating block to release DNA from any bound *C*. *jejuni* cells. The tubes were centrifuged for 2 min at 21,800 g to sediment beads and cell debris leaving DNA in the supernatant. *C*. *jejuni* DNA in the supernatant was quantified by qPCR. Subsequently, to evaluate the specificity of the IMS process, IMS was repeated to capture *C*. *jejuni* cells from a mixed sample containing *C*. *jejuni*, *C*. *coli*, and *C*. *lari* (each present at 10^4^ cfu/ml), before quantification of the captured *C*. *jejuni*, *C*. *coli*, and *C*. *lari* by qPCR. *C*. *jejuni* was quantified by qPCR targeting the *hipOa* gene (Keramas et al. 2003) using primers CGTGCAGATATGGATGCTTTG and TGCAGCAAGCAATAAAGAAGTAG. *C*. *coli* was quantified by qPCR targeting the *glyAb* gene (Vondrakova 2014) using primers GCGAGTGCTTATGCTCGTATTA and CCAGCAATGTGTGCAATGTC. *C*. *lari* was quantified by qPCR targeting the *pepTc* gene (Vondrakova 2014) using primers GAGCTGATGATAAAGCTGCTATTG and CACCTCTTAGGCCTTGTTCTT. All of the qPCR reactions were performed in triplicate. Input and captured cells were quantified against standard curves and % recovery of cells by IMS was calculated.

## Results

### Successful immunization of rabbits with *C*. *jejuni* whole cell antigen and construction of scFv phage display library

**Fig. 1:**
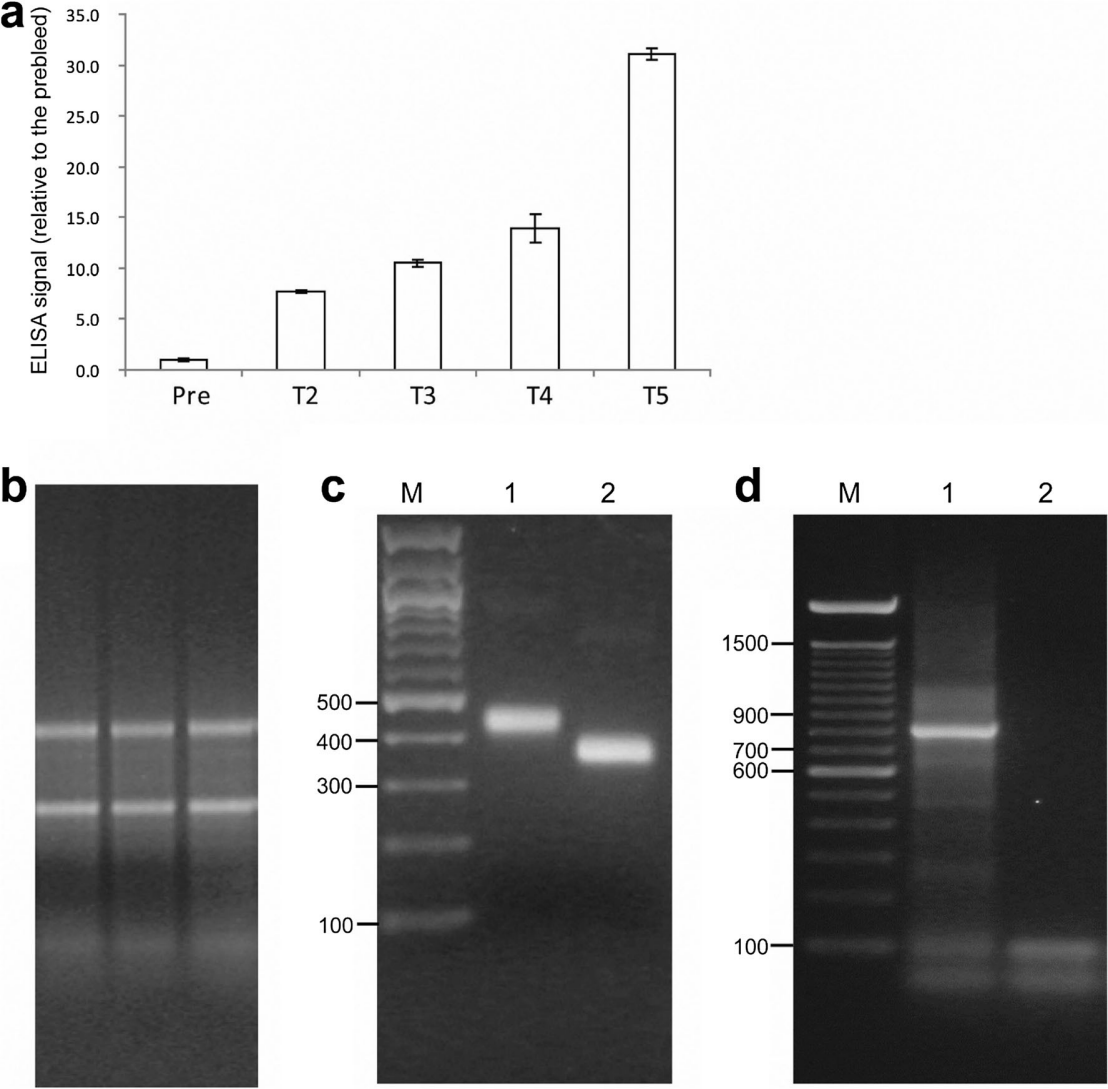
Successful immunization of a rabbit with *C*. *jejuni* WCA and amplification of scFv cDNA fragments from spleen of the rabbit. **a** ELISA signal against *C*. *jejuni* cells, using pre-bleed and antisera from the rabbit after two (T2), three (T3), four (T4), and five (T5) immunizations. Each column represents the mean fold difference in ELISA signal from pre-bleed. The error bars represent standard deviation of the signals in the triplicate wells for each antibody. **b** Agarose gel image of the three total RNA samples purified from the spleen of the rabbit immunized with *C*. *jejuni* WCA. The sharp rRNA bands show that the RNA samples had no degradation. **c** Agarose gel image of purified cDNA fragments for V_H_ and V_L_ chains amplified from immune rabbit spleen. PCR products using nine pairs of V_K_ and 1 pair of V_λ_ primers were pooled together to purify the V_L_ fragment. PCR products using four pairs of V_H_ primers were pooled together to purify the V_H_ fragment. Lane M = 100 bp ladder; bp size of some bands is shown on the left of the image. Lane 1 = ~ 450 bp V_H_ cDNA, lane 2 = ~ 350 bp V_L_ cDNA. **d** Agarose gel image of purified full-length scFv cDNA fragment after merging the V_L_ and V_H_ fragments by overlap extension PCR. Lane M = 100 bp ladder. Lane 1 = ~ 800 bp scFv cDNA fragment, lane 2 = non-template PCR control

After the immunization course was completed for two New Zealand White rabbits, a substantial increase in anti-*C*. *jejuni* IgG production was observed in one of the rabbits (Fig. S1; Fig. 1a) and spleen and bone marrow were harvested from this rabbit. Total RNA extracted from the spleen (Fig. 1b) was used to synthesize the first-strand complementary DNA (cDNA) from which individual V_L_- and V_H_-chain region genes of approximately 350 and 400 bp, respectively, were amplified by PCR (Fig. S1, S2; Fig. 1c). The V_L_ and V_H_ chains were randomly joined by overlap extension PCR through a linker sequence to construct the 800 bp cDNA encoding full-length scFv fragments (Fig. S1, Fig. 1d).

Following digestion with *Sfi*I, scFv constructs of approximately 750 bp in size were ligated into the *Sfi*I sites of a 3.4 kb linearized, dephosphorylated pComb3XSS phagemid vector. A library of phage particles displaying scFv on the surface, comprised of 6.8 × 10^7^ independent transformants, was produced by successful transformation of the ligation into *E*. *coli* ER2738 electrocompetent cells and rescuing the library by infecting with helper phage M13 K07. The efficiency of library construction, determined by randomly selecting 93 colonies from the titered library and performing colony PCR, showed that the positive rate of transformants containing cDNA in the phagemid was 96%.

### Screening and characterization of scFv-displaying particles which can strongly and specifically recognize *C*. *jejuni* cells

**Fig. 2:**
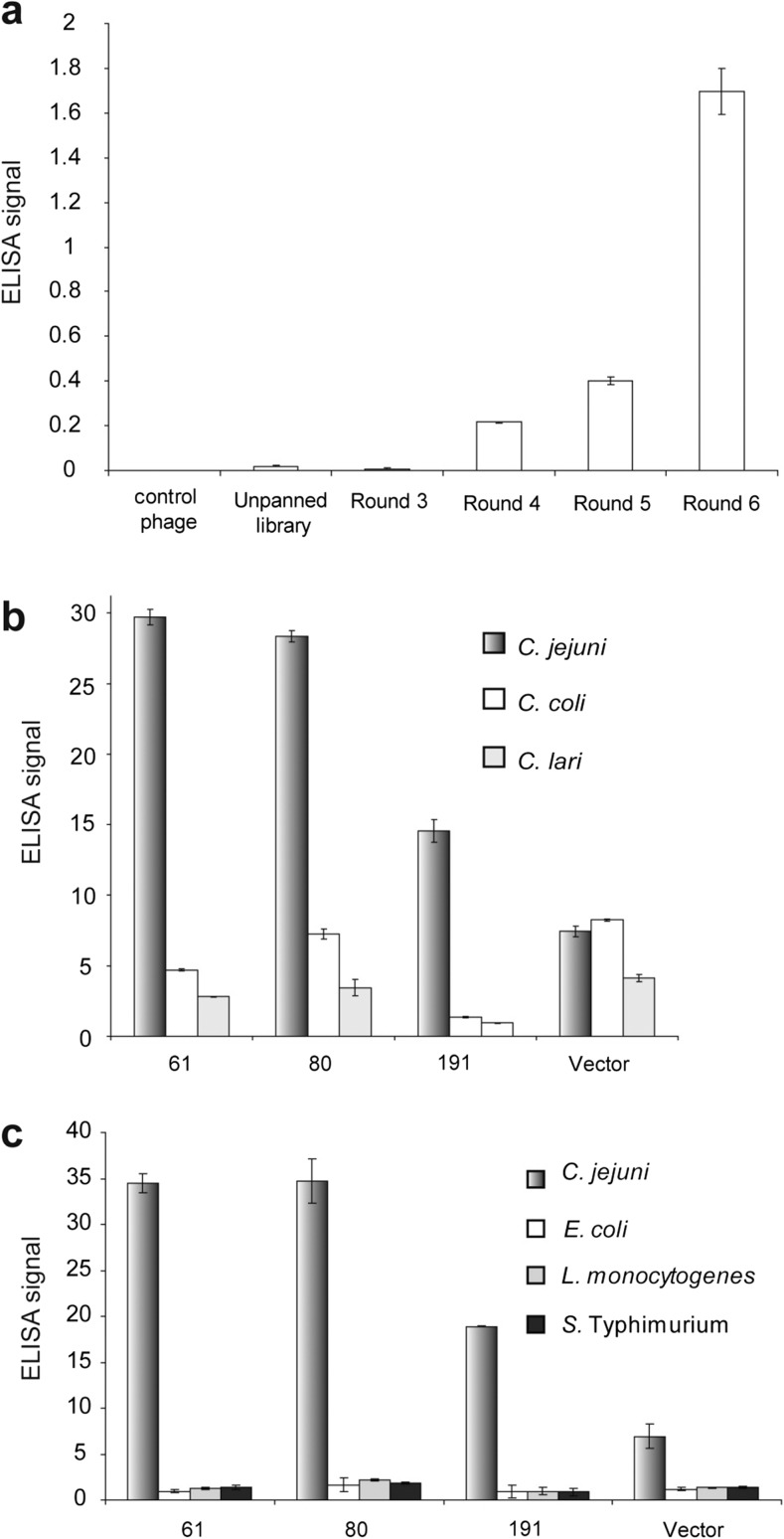
Enrichment of *C*. *jejuni*-binding scFv-displaying phages by biopanning and characterization of selected phage clones for binding specificity. In **a**–**c** equal amount of cells and phage particle were used in each experiment. The bound phage particles were detected using HRP-conjugated anti-M13 antibody and the substrate ABTS. **a** ELISA assay to test binding activity to *C*. *jejuni* cell, using scFv-displaying phage pools that resulted from different rounds of biopanning. The control phage is the non-displaying phage. Signal reading at 415 nm is shown in the *Y*-axis. **b** The three best scFv-displaying phage clones (scFv 61, scFv 80, scFv 191) were tested in ELISA assay for their binding activity to *C*. *jejuni* and other *Campylobacter* spp. **c** The three best scFv-displaying phage clones were tested in ELISA assay, comparing their binding activity to *C*. *jejuni* and three non-*Campylobacter* bacterial cells. In **b**, **c**, the signal reading at 415 nm of the cell-coated wells was normalized against that in the uncoated wells. Each column and error bar in **a**–**c** represents the mean and standard deviation of triplicates of reactions

After six rounds of biopanning, including double recognition in first round, the effectiveness of the enrichment in phage pools from different rounds was verified by indirect phage-binding ELISA assay (Fig. 2a). The unpanned library showed slight signal compared to the control phage that did not display scFv, demonstrating that the constructed library contained phage clones that recognized *C*. *jejuni* WCA. However, no significant enrichment of antibodies was found in phage pools after three rounds of biopanning. Progression in enrichment of positive phage clones was detected gradually with increase in stringency of washes and once high stringency washing was adopted from round 4 to round 6, an 8.5-fold enrichment of phage particles in phage pools was achieved, demonstrated by an increase in OD_415_ from 0.2 to1.7.

One hundred thirty-five individual clones randomly selected from titered output phages after round 6 were checked for diversity of the scFv cDNA sequences by PCR followed by digestion with restriction enzyme *Mva* I. Analysis of the digestion patterns revealed 47 unique groups (data not shown). A representative clone from each group was analyzed for binding activity and specificity to freshly prepared (non-irradiated) *C*. *jejuni*, *C*. *coli*, and *C*. *lari* WCAs using phage ELISA. Clones with signals at least two times greater than that of the empty vector control were considered to be binding *Campylobacter* cells. This testing revealed 12 clones which strongly recognized *C*. *jejuni* (Fig. S3); 3 of which (61, 80, and 191, from different *Mva* I digestion groups) specifically recognized *C*. *jejuni* and not the other 2 *Campylobacter* spp. (*C*. *coli* and *C*. *lari*) (Fig. 2b) or other unrelated foodborne pathogens (*L*. *monocytogenes*, *S*. Typhimurium, and *E*. *coli*) (Fig. 2c) tested.

### Expression and purification of anti-*C*. *jejuni* scFv monoclonal antibodies

**Fig. 3:**
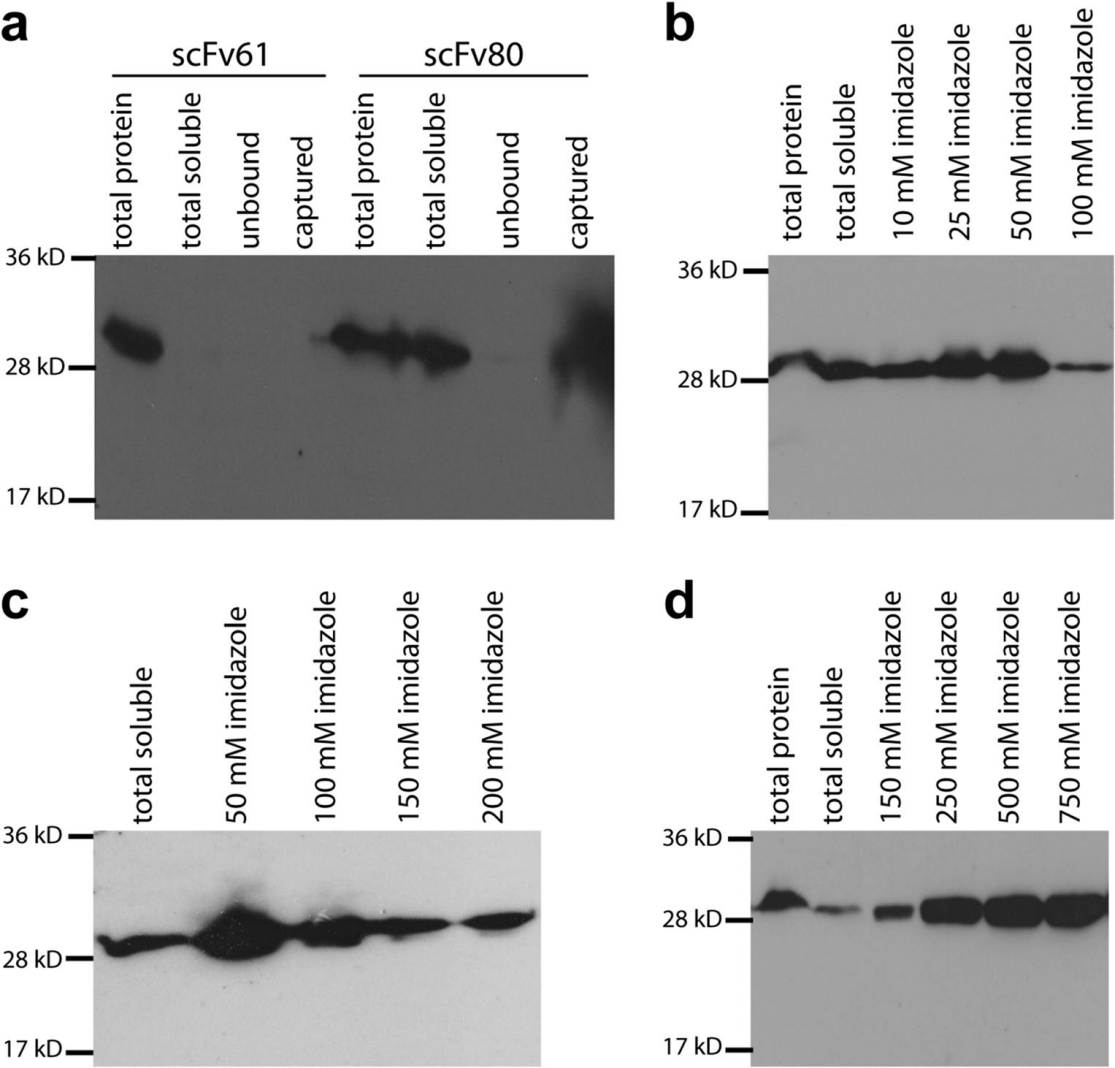
Expression and purification of *C*. *jejuni*-specific scFv antibodies**. a** Western blot of scFv61 and scFv80, expressed in *E*. *coli* and captured by affinity beads. Both antibodies have 6xHis tag at their C-terminus and were detected using His-probe. Nickel beads were used to capture the soluble antibodies. **b** Western blot of scFv80 expressed and purified using different imidazole concentrations in the binding buffer. **c** Western blot of scFv80 expressed and purified using different imidazole concentrations in the washing buffer. **d** Western blot of scFv80 expressed and purified using different imidazole concentrations in the elution buffer

Clones scFv61 and scFv80 were selected for expression of antibody fragments not fused to phage particles. Recombinant pComb3XSS phagemid DNA of scFv61 and scFv80 was isolated from *E*. *coli* ER2738 cells, transformed into TOP10F′ cells (a nonsuppressor strain of *E*. *coli*) and confirmed by colony PCR. The transformed TOP10F′ cells were induced by IPTG to produce a protein band of ~ 30 kDa in total protein fractions of both scFv61 and scFv80 as revealed by Western blotting (Fig. 3a), demonstrating successful expression of both antibodies. However, only scFv80 was shown to be expressed in a soluble form, as shown by the presence of the ~ 30 kDa protein band in the total soluble fraction of scFv80 but not that of scFv61. While both antibodies were fused with a poly histidine tag (His6-tag) at the C-terminus, the presence of a ~ 30 kDa band in the captured fraction of scFv80 only clearly shows that the soluble scFv80 protein readily bound to Ni-NTA resin and was successfully eluted. In contrast, absence of protein band in the captured fraction of scFv61 confirmed that it was expressed as insoluble protein, which could not be captured by beads.

To maximize the purity of the scFv80 when using HisPur Ni-NTA affinity Resin, concentration of imidazole in binding, washing, and elution buffers was optimized. Increasing the concentration of imidazole in the binding buffer to 50 mM was observed to give the highest stringency with optimum binding of scFv80 to the beads, while 100 mM of imidazole significantly reduced capturing of the antibody (Fig. 3b). Analysis of the impact of different concentrations of imidazole (50, 100, 150, and 200 mM) in the washing buffer after binding scFv80 antibody in 50 mM of imidazole showed that washes in 50 mM imidazole retained the highest yield of scFv80 on beads, whereas imidazole concentrations above 50 mM significantly washed off the antibody from the beads (Fig. 3c). Elution efficiency of scFv80 antibody in elution buffer containing 150, 250, 500, or 750 mM of imidazole from beads that had been bound and washed with the optimized 50 mM imidazole respectively showed that elution efficiency increased significantly from 150 to 250 mM of imidazole and no further significant increase in elution efficiency was observed beyond 250 mM of imidazole (Fig. 3d). Therefore, the optimal concentrations of imidazole for producing pure levels of scFv80 antibody were 50 mM in binding buffer, 50 mM in washing buffer, and 250 mM in elution buffer.

### Binding characteristics of purified anti-*C*. *jejuni* scFv80 antibody to bacterial cells

**Fig. 4:**
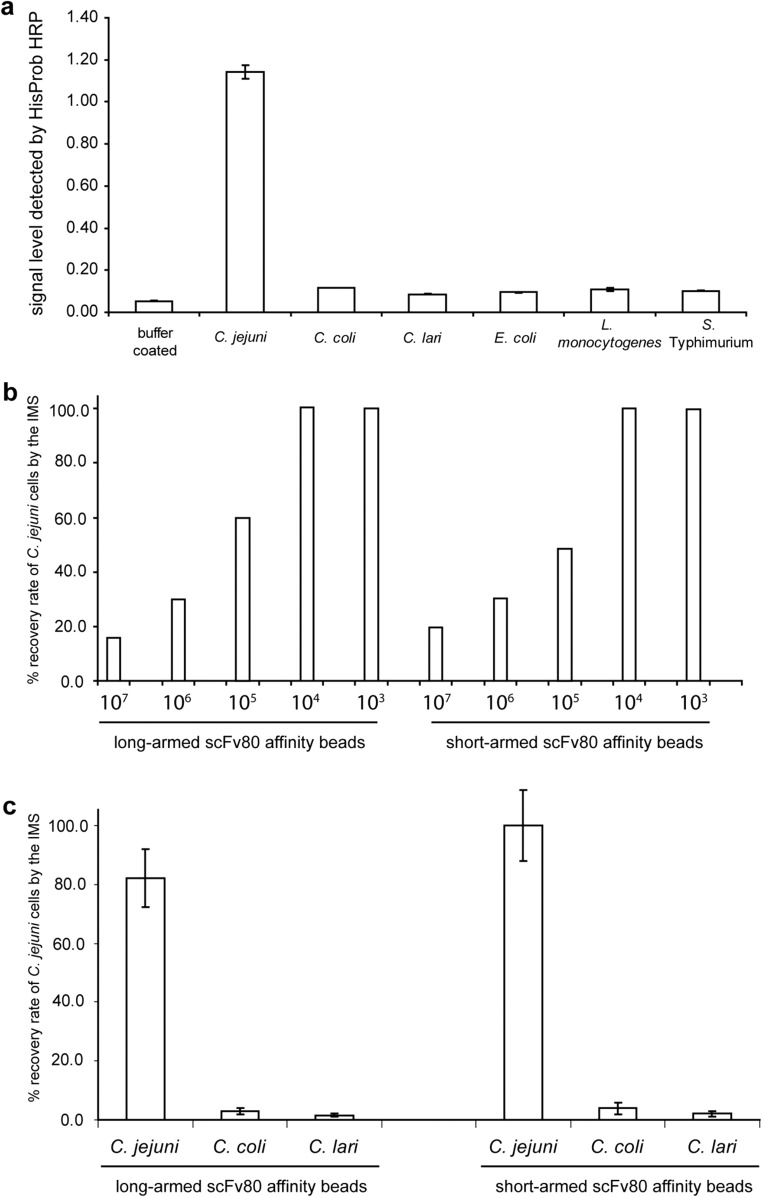
Purified scFv80 antibody specifically recognizes *C*. *jejuni* cells. **a** In the ELISA assay, purified scFv80 was used to detect *C*. *jejuni* and other foodborne pathogens. The same amount of bacterial cells and scFv80 antibody was used in all the ELISA wells. Bound scFv80 was detected by HisProbe-HRP Conjugate and the substrate TMB (E). Signal read at 450 nm was shown in the *Y*-axis. Each column represents the mean and standard deviation of signals in three wells. **b** Recovery of *C*. *jejuni* by IMS from solutions containing different cell concentrations (10^3^–10^7^ cfu/ml) using scFv80 coated beads. Cell concentrations are labeled below each column. Recovery was calculated according to qPCR quantification data of the captured and input cells. **c** Recovery of *Campylobacter* cells by IMS using scFv80 coated beads from a mixed culture of *C*. *jejuni*, *C*. *coli*, and *C*. *lari* (each present at 10^4^ cfu/ml). qPCR reactions specific to *C*. *jejuni*, *C*. *coli*, or *C*. *lari* were used to quantify the input and captured cells before calculating the recovery rate of each species

A large-scale culture of scFv80 antibody was purified using the optimized imidazole concentrations. The concentration of purified scFv80 was estimated to be 180 ng/μl by ELISA using commercial His-tagged cTnI as a standard. The purified scFv80 antibody was tested for its binding specificity to *C*. *jejuni* and other non-target bacteria in an ELISA assay. This assay clearly demonstrated that the purified scFv80 specifically recognized *C*. *jejuni* cells but did not bind to either its relatives (*C*. *coli* and *C*. *lari*), or the three other unrelated foodborne pathogens (*E*. *coli*, *S*. Typhimurium, or *L*. *monocytogenes*), even though these cells were exposed to the antibody at a high concentration of 1 × 10^8^ cells/well (Fig. 4a).

### Evaluation of purified scFv80 in IMS procedure to capture *C*. *jejuni* cells

The capacity of purified scFv80 antibody as a ligand to capture *C*. *jejuni* cells by IMS was tested using long-arm and short-arm BcMag™ tosylactivated paramagnetic beads that had been covalently coated with the antibody. The captured cells were quantified by qPCR. When sensitivity of the IMS in recovering *C*. *jejuni* cells from samples containing 10^3^ to 10^7^ cells/ml was assessed, both long-arm and short-arm tosylactivated beads coated with scFv80 were found to be able to capture 100% of *C*. *jejuni* cells from dilutions containing 10^3^ to 10^4^ cells/ml (Fig. 4b). It was observed that when the cell input was higher than 10^4^ cells/ml, the recovery rate of *C*. *jejuni* by the beads decreased, ranging from 16 to 60% (Fig. 4b), which suggested that the coated beads had a maximum binding capacity.

In biological samples, *C*. *jejuni* could be present along with other cells, so to test if scFv80-coated beads could specifically recover *C*. *jejuni* from mixed cell suspensions, IMS was performed on a sample containing *C*. *jejuni*, *C*. *lari*, and *C*. *coli*, all at 10^4^ cells/ml. Recovered cells were quantified by qPCRs targeting genes specific to *C*. *jejuni*, *C*. *lari*, and *C*. *coli*. Figure 4c shows that in this IMS, the short-arm beads recovered 100% *C*. *jejuni*, whereas the long-arm beads recovered 82%. The short-arm scFv80-coated beads performed better than long-arm scFv80-coated beads, although both beads were very specific for *C*. *jejuni*, capturing only 1.5–4% of either *C*. *coli* or *C*. *lari* cells*.*

## Discussion

Campylobacteriosis, especially caused by *C*. *jejuni*, is the most common bacterial cause of human gastroenteritis worldwide (Silva et al. 2011; Tram et al. 2012). Poultry is the main reservoir of *C*. *jejuni* (Skarp et al. 2016) and most human infections are caused by consuming undercooked poultry or mishandling of contaminated poultry products (Silva et al. 2011). Effective monitoring of *C*. *jejuni* in chickens prior to slaughter is crucial to tackling *Campylobacteriosis*, but better detection methods are urgently needed for this purpose.

Here, we present the results of construction of a scFv-phage displaying library derived from a *C*. *jejuni* immunized rabbit, and selection of phage clones displaying scFv antibodies recognizing *C*. *jejuni* cell by phage display biopanning. In addition, we describe development of an IMS method employing one of the *C*. *jejuni*-specific scFv antibodies produced, to capture and concentrate *C*. *jejuni* from test samples. This IMS method was subsequently combined with a qPCR method to achieve rapid detection and quantitation of *C*. *jejuni*. We demonstrate that the resultant IMS-qPCR method could potentially be suitable for *C*. *jejuni* monitoring purposes, subject to some further optimization.

In order to produce novel recombinant scFv antibodies specific for *C*. *jejuni* cells, two rabbits were immunized with *C*. *jejuni* whole cells that had been subjected to gamma radiation rather than heat-inactivation before use. Irradiation kills the bacterial cells while maintaining the integrity of cell surface antigens, unlike heating which is liable to denature some or all of these surface antigens (Stewart et al. 2012). Once a high serum antibody titer against *C*. *jejuni* had been reached in one of the immunized rabbits, indicating that there was enrichment for antigen-specific antibodies, the rabbit spleen was harvested for RNA extraction. Either spleen or bone marrow from the immune-responsive rabbit could have been selected as the starting point for recombinant antibody production. We selected spleen as our starting material because for high titer animals, like the one in this study, it has been shown that V genes encoding antigen-specific antibodies dominate the large diverse B lymphocytes in the spleen indicating that total splenocytes can be used in place of bone marrow plasma cells for antibody discovery (Saggy et al. 2012). The V_L_ and V_H_ chain genes were separately amplified from the spleen cDNA along with the short linker sequence through which the individual cDNA fragments were randomly joined by overlap extension PCR to produce full-length scFv cDNA fragments (Barbas et al. 2001). The short linker joining V_H_ and V_L_ in scFv maintains the antigen-binding sites, with enhanced avidity for the specific antigen, as previously noted by Turner et al. (1997) and Singh et al. (2010). According to Barbas et al. (2001), the isolation of specific antibodies from a cloned immunological repertoire requires a large, diverse, and complex library in the range of 10^7^ to 10^8^ independent transformants, as well as an efficient selection process. In this study, when cloned into the phagemid vector pComb3XSS which had been dephosphorylated in order to increase the proportion of transformants containing the cDNA fragments, the scFv genes resulted in a combinatorial library with 6.8 × 10^7^ clones. This was considered to have sufficient complexity to proceed to phage display. The scFv antibodies were expressed as fusions to the gene III coat protein (pIII) of the phage surface, as first described by Smith (1985), which made it possible for them to be screened by successive rounds of biopanning against the *C*. *jejuni* WCA (Barbas et al. 2001) to enrich clones expressing scFv antibodies strongly recognizing *C*. *jejuni* cells.

ELISA analysis of the outcome phages of the first three rounds of biopanning did not show significant enrichment of *C*. *jejuni* binders (Fig. 2a). However, some evidence of enrichment began to be observed after round 4, doubled after round 5, and then showed a marked increase after round 6 (Fig. 2a). As the stringency of the selection conditions increased with successive rounds of biopanning, more scFv-displaying phage clones with better binding capabilities were successfully selected, resulting in library enrichment at round 6 of biopanning. After round 6, 135 clones were randomly selected to amplify the scFv cDNA and subjected to *Mva* I restriction digestion; the digestion patterns were used to quickly test sequence diversity of scFv cDNA among the large numbers of clones (Barbas et al. 2001).

Two phage clones, 61 and 80, were selected to express scFv antibodies without pIII. pComb3XSS contains the amber codon such that when transformed into a non-suppressor bacterial strain, it can express soluble protein without coat protein III (Barbas et al. 2001). These scFv antibodies are fused with a poly histidine tag (His6-tag) and hemagglutinin tag (HA-tag) at their C-terminus which aids affinity purification and immuno-detection of the antibody proteins by Western blotting. Phagemid DNA isolated from clones 61 and 80 was transformed into the non-suppressor *E*. *coli* TOP10F′ cells. Protein expression was induced by IPTG at 37 °C for 18 h. scFv80 was expressed as a soluble protein (Fig. 3a); a pure, single scFv80 protein band, of size ~ 30 kDa, was observed on SDS-PAGE gel and by Western blotting. In contrast, scFv61 was expressed as an insoluble protein. The high-level expression of scFv61 in TOP10F′ cells may have led to formation of highly aggregated protein commonly referred to as inclusion bodies (Palmer and Wingfield 2012), resulting in it being insoluble. Several alternative strategies to generate soluble scFv61 were tried, including expressing the protein at varying temperatures in TOP10F′ cells, supplementing with glucose and sorbitol, and expression in a different non-suppressor strain, *E*. *coli* BL-21-A in the presence of 0.2% (*w*/*v*) arabinose, but all have proved unsuccessful to date.

The specificity of the purified, soluble *C*. *jejuni*-specific scFv80 antibody was assessed by ELISA. The recombinant antibody was confirmed to have retained its high binding capability for *C*. *jejuni* WCA when not expressed on the phage and it still did not cross-react with two other *Campylobacter* spp. or three other unrelated foodborne bacterial pathogens (Fig. 4a). These results indicated that scFv80 was very specific for *C*. *jejuni* and, hence, potentially suitable to be incorporated into a novel detection approach for this foodborne pathogen*.* IMS linked with qPCR was our novel detection approach of choice because IMS offers the possibility of selectively extracting *C*. *jejuni* cells from heterogeneous suspensions, such as those that are encountered in food, clinical specimens, and feces, and qPCR offers the possibility of rapid, specific, and quantitative detection of the captured *C*. *jejuni* cells assuming appropriate primers are selected. Therefore, purified scFv80 antibody was coated onto tosylactivated magnetic beads and suitable primers were selected/designed for qPCR detection; targeting the HipO gene, encoding hippuricase, that is absent in all *Campylobacter* species except *C*. *jejuni* (Keramas et al. 2003), and the glyA and pepT genes in *C*. *coli* and *C*. *lari,* respectively (Vondrakova 2014).

Immunomagnetic separation has been successfully used to capture foodborne *Salmonella* spp. and *Listeria monocytogenes* using beads coated with phage display-derived peptides (Carnazza et al. 2007; Morton et al., 2013a, b), or beads coated with a phage display-derived single-chain antibody for *Listeria monocytogenes* (Paoli et al. 2007). IMS methods for *C*. *jejuni* involving polyclonal and monoclonal antibodies have previously been reported (Yu et al. 2001; Tram et al. 2012). To our knowledge, this study is the first to assess the IMS potential of a recombinant scFv antibody specific for *C*. *jejuni*. The novel recombinant scFv80 antibody was evaluated for IMS by coating two types of tosylactivated paramagnetic beads (‘short-arm’ and ‘long arm’); these were both shown to be capable of capturing 100% *C*. *jejuni* cells from a pure culture containing 1000 to 10,000 cfu/ml (Fig. 4b). We did not test recovery of cells below 1000 cfu/ml. However, we have tested the sensitivity of the qPCR step and found that it can detect as low as five cells (data not shown). Therefore, we are confident the limit of detection (LOD) of the IMS can be further reduced. Percentage recovery from high concentrations of *C*. *jejuni* by the IMS was low (Fig. 4b). However, this is not an issue in terms of potential application of the beads in real life sample because rarely would biological samples have such high levels of *C*. *jejuni* present. The long- and short-arm beads both specifically and selectively captured 82 and 100% *C*. *jejuni* cells, respectively, from a mixed suspension containing 10,000 cfu/ml of each of *C*. *jejuni*, *C*. *coli*, and *C*. lari (Fig. 4c). In this study, 1 ml of cell samples was used in IMS. However, real biological samples are often in volumes of 50 to 250 ml. A centrifugation step could be introduced to concentrate the cells into 1 ml before applying the IMS to this concentrated sample. This experiment demonstrated that the IMS procedure using recombinant monoclonal scFv80 antibody derived from the spleen of a *C*. *jejuni* immunized rabbit specifically captured *C*. *jejuni*. To our knowledge, our work represents the first attempt to use recombinant scFv antibodies in IMS for capturing *Campylobacter* spp. to date. The IMS-qPCR method was able to specifically detect 1000 cfu/ml *C*. *jejuni* in just under 3 h. With some further optimization of the IMS, it may be possible to improve on the detection sensitivity.

In conclusion, a novel anti-*C*. *jejuni* recombinant scFv antibody, scFv80, demonstrating strong and specific binding to *C*. *jejuni* cells was successfully generated from an immune spleen combinatorial library by phage display. When this novel recombinant monoclonal scFv80 antibody was coated onto BcMag™ long-arm and short-arm tosylactivated magnetic beads, and IMS was carried out in combination with qPCR as the endpoint detection method, the resultant IMS-qPCR assay indicated the specific capture of 82–100% *C*. *jejuni* cells, from a mixed culture containing *C*. *jejuni*, *C. coli*, and *C. lari.* The scFv80 antibody-based IMS-qPCR method merits further development and optimization, as it could potentially reduce the current detection time for *C*. *jejuni* from 3 to 5 days to just under 3 h without the need for a culture enrichment step.

## Electronic supplementary material

ESM 1 (PDF 412 kb)
