# Supplementary material for: Generation and characterization of a novel recombinant scFv antibody specific for Campylobacter jejuni
Source: Appl Microbiol Biotechnol. 2018 Apr 7;102(11):4873–85. doi: 10.1007/s00253-018-8949-x (PMC5953994; doi:10.1007/s00253-018-8949-x)
Supplement: Supplementary file 1 — (PDF 412 kb) [file 253_2018_8949_MOESM_ESM.pdf]

Applied Microbiology and Biotechnology

Supplementary Material

Generation and characterisation of a novel recombinant scFv antibody specific for *Campylobacter jejuni*

Ruramayi M. Nzuma, Fuquan Liu and Irene R. Grant

Institute for Global Food Security, School of Biological Sciences, Queen's University Belfast, Belfast, BT9 7BL, Northern Ireland, United Kingdom

Corresponding authors: Dr Irene Grant, Tel: +44 28 9097 2109, email: i.grant@qub.ac.uk.

Dr Fuquan Liu, Tel: +44 28 9097 2477, email: f.liu@qub.ac.uk

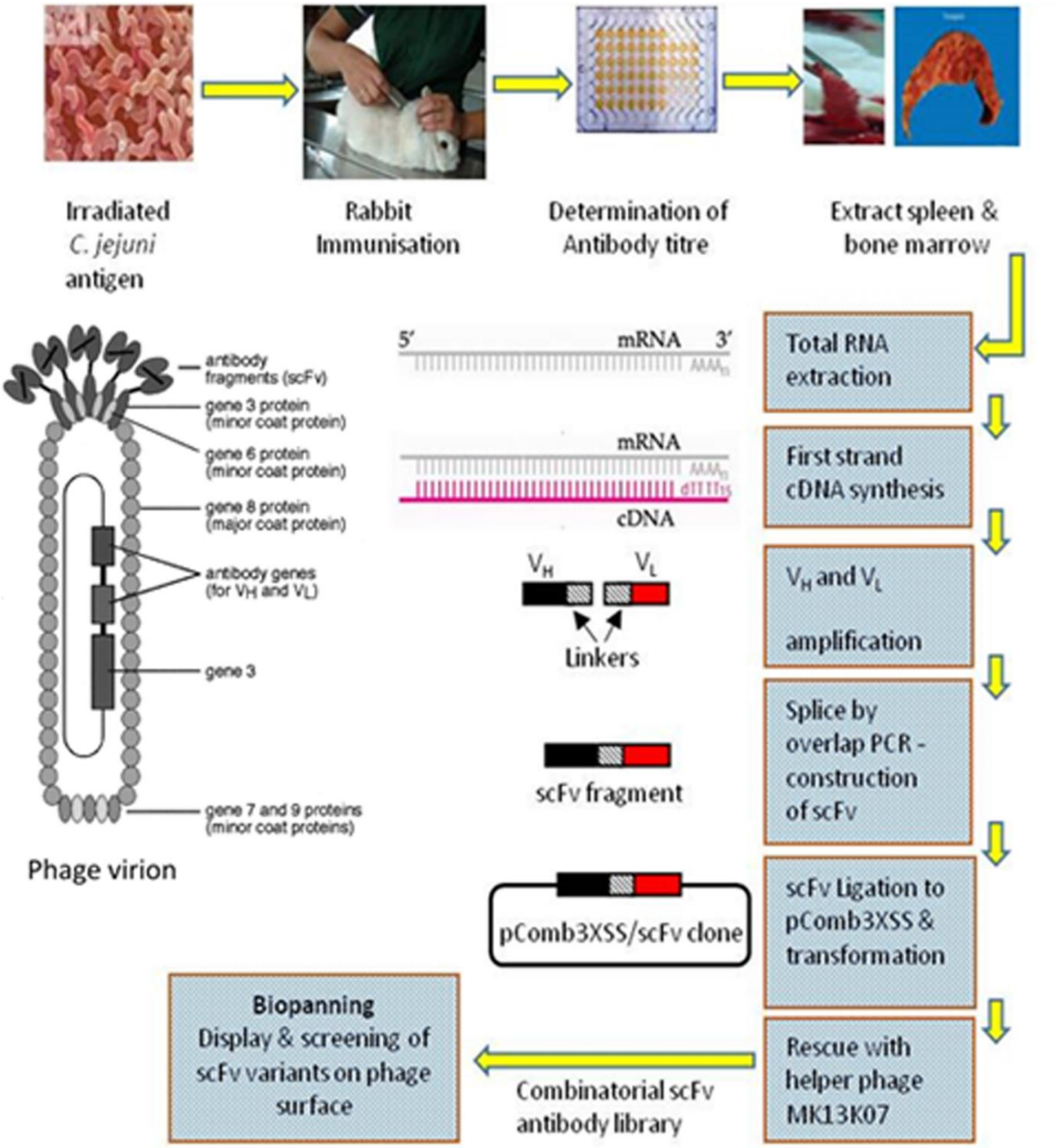

Fig. S1. Flowchart of construction of the scFv-displaying phage library from immunized rabbit. The rabbit was immunized with γ-irridiated *C. jenuni* whole cell. Total RNA was extracted from spleen of the immune rabbit. The cDNA fragments of V_H and V_L were amplified from the cDNA, fused into full length scFv cDNA fragments, ligated to the phagmid pComb3XSS and transformed into *E coli* ER2738 to the cDNA library. scFv-displaying phage library was generated by infecting the cDNA library with the helper phage MK13K07. The scFv antibody is displayed on the surface of the phage as fusions to the gene 3 protein. The resulted phage library was screened against *C. jenuni* whole cell through 6 rounds of biopanning.

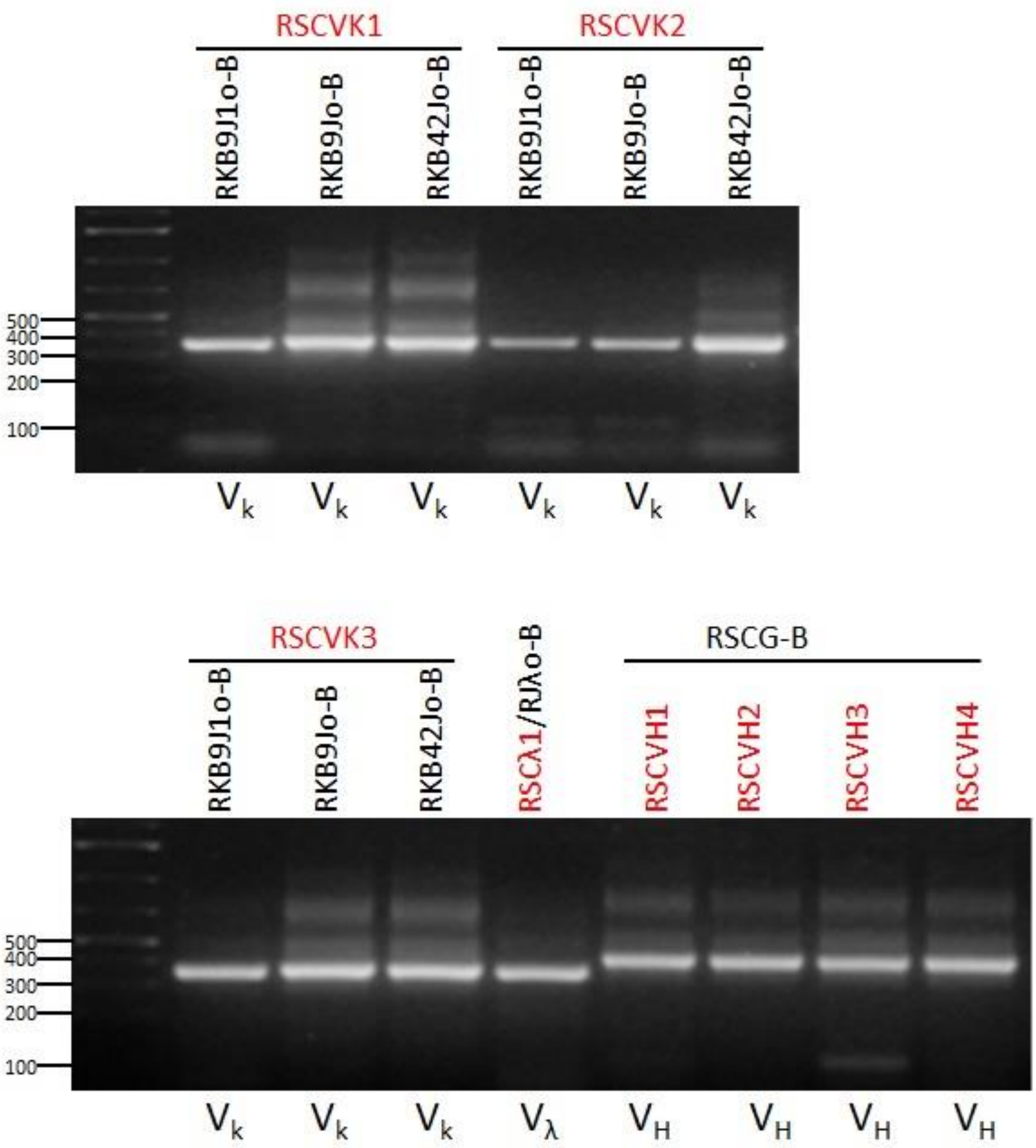

Fig. S2. Gel images of the PCR products of the VL and VH cDNA fragments amplified from the spleen of the immune rabbit. Primer names are labelled on the top of the lanes, forward primers in red. Nature of the cDNA fragments are labelled below the lanes. 10 pairs of primers were used to amplify the 350 bp VL cDNA (including 9 pairs for Vk and 1 pair for Vλ). Four pairs of primers were used to amplify the 450 bp VH cDNA. The 1st lane of each gel has 100 bp MW ladder, five bands are labelled in bp on the left of the image.

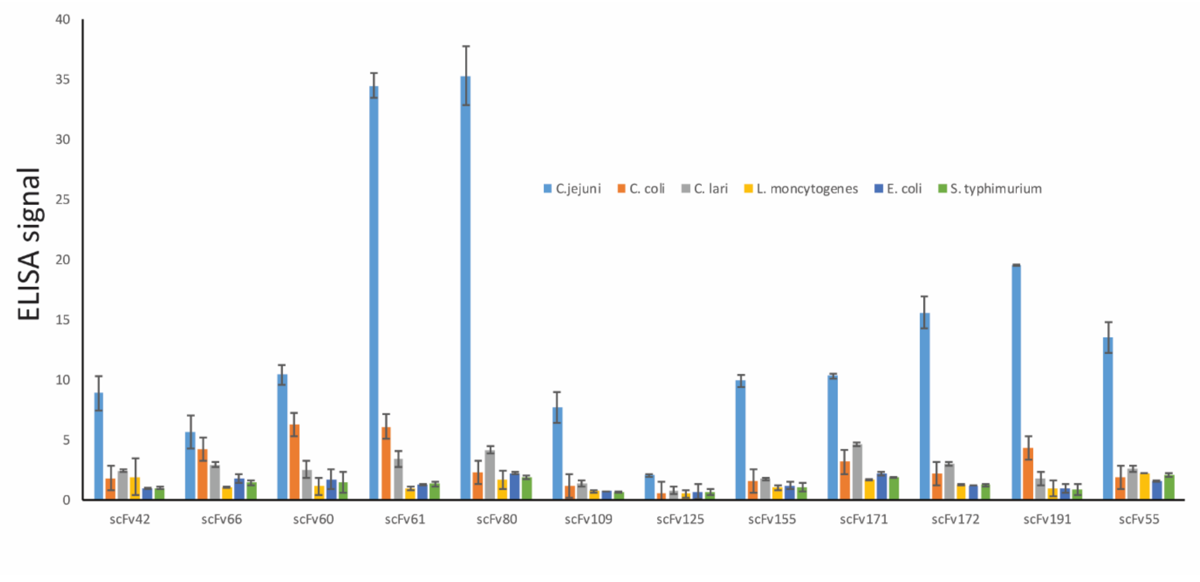

Fig. S3. ELISA assay to test binding activity of the 12 phage clones to three *Campylobacter* spp. and three other three food borne pathogenic bacteria. Equal amount of bacteria cells was coated in each well. Equal amount of phage was also used in all the ELISA wells. The signal reading at 415 nm of the cell-coated wells was normalized against that in the uncoated wells. Each column represents the mean and standard deviation of the normalized reading from triplicate wells.
